# Choice of Teeth for Extraction for the Purpose of Uniformity

**Published:** 1885-05

**Authors:** 


### Choice of Teeth for Extraction for the Purpose of Uniformity.

The *Weekly Medical Review*, Nov. 15, 1884, says:

The physician is not always consulted relative to the best course to adopt in regard to the teeth, but he cannot afford to be ignorant of the peculiarities of this important part of the anatomy. When the teeth are too much crowded in the jaw, it is frequently desirable for æsthetic and hygienic purposes to draw some of them, and the question which to draw when there is a choice has, it appears, been a constant source of discussion among dentists. Dr. Perry, in order to throw some light on the subject, has tabulated 7,277 extractions for disease, and finds that 2,823 of these were first permanent molars, 737 were first bicuspids, and 944 were second bicuspids. As the statistics show that more first molars are lost than bicuspids, we should, as a general rule, take out the first molar where the choice must be, as is usually the case, between this and a bicuspid.

